# Neurological post-COVID syndrome is associated with substantial impairment of verbal short-term and working memory

**DOI:** 10.1038/s41598-025-85919-x

**Published:** 2025-01-11

**Authors:** Jeyanthan Charles James, Hannah Schulze, Nadine Siems, Christian Prehn, Daniel R. Quast, Nadine Trampe, Ralf Gold, Simon Faissner

**Affiliations:** 1 Department of Neurology, St. Josef-Hospital, Ruhr-University Bochum, Gudrunstr. 56, 44791 Bochum, Germany; 2 Department of Internal Medicine, St. Josef-Hospital, Ruhr-University Bochum, Gudrunstr. 56, 44791 Bochum, Germany

**Keywords:** Neurological Post-COVID syndrome, Post-COVID syndrome, Long-COVID syndrome, SARS-CoV-2, Neurology, Neurological manifestations

## Abstract

A substantial proportion of patients suffer from Post-COVID Syndrome (PCS) with fatigue and impairment of memory and concentration being the most important symptoms. We here set out to perform in-depth neuropsychological assessment of PCS patients referred to the Neurologic PCS clinic compared to patients without sequelae after COVID-19 (non-PCS) and healthy controls (HC) to decipher the most prevalent cognitive deficits. We included *n* = 60 PCS patients with neurologic symptoms, *n* = 15 non-PCS patients and *n* = 15 healthy controls. Basic socioeconomic data and subjective complaints were recorded. This was followed by a detailed neuropsychological test battery, including assessments of general orientation, motor and cognitive fatigue, screening of depressive and anxiety symptoms, information processing speed, concentration, visuomotor processing speed, attention, verbal short-term and working memory, cognitive flexibility, semantic and phonematic word fluency, as well as verbal and visual memory functions. Neurologic PCS patients had more complaints with significantly higher fatigue scores as well as higher levels of depressive and anxiety symptoms compared to Non-PCS and HC. Deep neuropsychological assessment showed that neurologic PCS patients performed worse in a general screening of cognitive deficits compared to HC. Neurologic PCS patients showed impaired mental flexibility as an executive subfunction, verbal short-term memory, working memory and general reactivity (prolonged reaction time). Multiple regression showed fatigue affected processing speed; depression did not. Self-reported cognitive deficits of patients with neurologic PCS including fatigue, concentration, and memory deficits, are well mirrored in impaired performance of cognitive domains of concentration and working memory. The present results should be considered to optimize treatment algorithms for therapy and rehabilitation programs of PCS patients with neurologic symptoms.

**Supplementary Information:**

The online version contains supplementary material available at 10.1038/s41598-025-85919-x.

## Introduction

In 2020, severe acute respiratory syndrome coronavirus type 2 (SARS-CoV-2) triggered one of the most severe pandemics in human history. The World Health Organization (WHO) estimates that by the end of 2023, approximately 773 million people had contracted acute SARS-CoV-2 infection, resulting in the multi-organ coronavirus disease 19 (COVID-19)^1^. Many of these affected patients continue to suffer from persistent or new symptoms, even 12 weeks after acute infection. This condition, in which symptoms persist or new symptoms develop at least three months after the acute infection and persist for at least two months without the finding of any causal relationship, is subsumed under the terms post COVID-19 condition^2^ or post-COVID syndrome (PCS)^3^. In the following, the term PCS refers to COVID-19 symptoms or new symptoms that cannot be attributed to any other etiology and persist more than 12 weeks after the acute COVID-19 infection. Symptoms commonly found and included in the symptom complex of PCS are hair loss, chronic kidney disease, thromboembolism, palpitations and chest pain, headache, cognitive impairment, post-traumatic stress disorder, sleep disturbances, anxiety and depressive symptoms, cough, shortness of breath, arthralgias, myalgias, and frequently and most importantly, chronic fatigue^4–10^. Neurological symptoms are particularly common, with fatigue and cognitive impairment being the most frequently documented^5,6,10,11^. Additionally, neurological and neuropsychiatric PCS encompasses symptoms such as dysosmia, sleep disorders, concentration deficits, amnesia, numbness, pain, depression, and anxiety^12^. The pathophysiologic mechanisms underlying PCS and its neurologic symptoms remain to be elucidated. However, regarding neuropathology, several competing mechanisms are hypothesized to explain this epiphenomenon. These include direct viral penetration, systemic inflammation with complement activation, microglia activation, activation of coagulation, oxidative stress, auto-immunity, and neurodegenerative processes^13–15^. From a clinical perspective, it is important to identify the most dominant cognitive deficits in these subjects and thus identify potential treatment targets for clinicians and basic researchers.

Therefore, the current study aims to perform in-depth neuropsychological assessment in patients with PCS to further elaborate on the neurological dimensions of PCS (for the sake of clarity and consistency, we will continue to use the abbreviation PCS throughout the text, tables, and graphs when referring to neurological PCS), subjects without clinical symptoms after COVID-19 (non-PCS) and healthy controls (HC).

## Methods

The study was conducted in accordance with the Declaration of Helsinki from 1975 and was approved by the local ethics committee of Ruhr-University Bochum (20-6827; 21-7423). Patients and controls were recruited at our specialized neurological Long-COVID clinic (Department of Neurology, Ruhr-University Bochum, St. Josef-Hospital, Bochum, Germany). Patient inclusion began in January 2021. Written informed consent was mandatory for participation. Patients older than 18 years with a history of COVID-19 infection (a positive PCR result was not required) who met the criteria for post-COVID syndrome, as defined earlier, were included in the PCS group. The persisting symptoms prompting their presentation at our outpatient clinic primarily focused on, but were not limited to, neurological issues. Subjects with a history of COVID-19 infection but without evidence of PCS were included in the non-PCS group. Subjects without history of COVID-19 infection were recruited as healthy controls. After inclusion in the study, sociodemographic data were collected.

### Self-assessment

Subjects completed a symptom self-assessment, including fatigue, muscle weakness, sleep disturbances, hair loss, olfactory disturbances, palpitations, joint pain, loss of appetite, taste disturbances, diarrhea, nausea, vomiting, chest pain, difficulty swallowing, skin rash, headache, muscle pain and memory disturbances. Additionally, subjects could report and specify other complaints or state that none of the mentioned symptoms were present. After completion of the self-assessment, neuropsychological testing was performed.

### Neuropsychological test battery

Neuropsychological examinations were performed by or under the supervision of two experienced neuropsychologists. The test battery was composed of a total of 11 different psychometric tests covering the cognitive domains general orientation, motor and cognitive fatigue, screening of depressive and anxiety symptoms, information processing speed, concentration, visuomotor processing speed, attention (tonic and phasic alertness), verbal short-term and working memory, cognitive flexibility, semantic and phonematic word fluency, as well as verbal and visual memory functions (Supplementary methods).

### Statistical analysis

The primary endpoint of the study was the number of subjects with PCS and substantial impairment of neurocognitive function as compared to non-PCS and HC. Data were presented as shown in the respective figure legends. Demographics, self-assessment, and clinical characteristics were compared. Depending on the psychometric procedure, raw scores, or standardized scores (z-scores) were calculated and used for analysis. Multiple regression analyses assessed the influence of group membership (PCS vs. non-PCS vs. HC), age, sex, education, and occupational status. FSMC total score, HADS-D, and HADS-A subscores were included as independent variables with statistical significance set at *p* < 0.05. Results were initially analyzed using the Kruskal-Wallis ANOVA. To adjust for multiple comparisons, the two-stage linear step-up procedure by Benjamini, Krieger, and Yekutieli (BKY) was applied, with *q* < 0.05 considered significant. All statistical analyses were performed using GraphPad Prism (version 10.3.1).

## Results

### Sociodemographic characteristics

A total of 90 subjects were included in the study, with a female proportion of 65.5% (*n* = 59). The PCS group consisted of *n* = 60 individuals (female proportion of 71.6%, *n* = 43) with neurologic symptoms, and the non-PCS and HC groups consisted of *n* = 15 individuals each (female proportion of 53.3%, *n* = 8). The mean age of all patients was 44 years with a median of 46 years (Table 1 and Fig. S1a). There were no differences between the groups regarding age.

**Table 1:** Sociodemographic data.

			PCS	NON-PCS	HC
Sex		Total number of participants	*n* = 60	*n* = 15	*n* = 15
		Female	*n* = 43 (71.7%)	*n* = 8 (53.3%)	*n* = 8 (53.3%)
		Male	*n* = 17 (28.3%)	*n* = 7 (46.7%)	*n* = 7 (46.7%)
Distribution of age		Median	46	40	30
		Mean	45	43	41
		SD (leated to mean value)	12	16	15
		Range	44	38	38
Professional and school education according to CASMIN classification	Distribution	No information (os = 0)	*n* = 0	*n* = 0	*n* = 0
		No degree (os = 1)	*n* = 0	*n* = 0	*n* = 0
		Secondary school diploma without vocational training (os = 2)	*n* = 0	*n* = 0	*n* = 0
		Secondary school diploma and vocational training (os = 3)	*n* = 6 (10%)	*n* = 0	*n* = 0
		Secondary school leaving certificate without vocational training (os = 4)	*n* = 0	*n* = 0	*n* = 0
		Secondary school leaving certificate and vocational training (os = 5)	*n* = 21 (35%)	*n* = 4 (26.7%)	*n* = 5 (33.3%)
		Advanced technical college certificate/ high school diploma without vocational training (os = 6)	*n* = 2 (3.3%)	*n* = 1 (6.7%)	*n* = 3 (20%)
		Technical college graduation/ high school diploma and vocational training (os = 7)	*n* = 11 (18.3%)	*n* = 2 (13.3%)	*n* = 1 (6.7%)
		University of applied sciences degree (os = 8)	*n* = 5 (8.3%)	*n* = 0	*n* = 3 (20%)
		University degree (os = 9)	*n* = 15 (25%)	*n* = 8 (53.3%)	*n* = 3 (20%)
	Descriptive statistics according to ordinal scales	median	7	9	6
		mean	6	7	7
		SD (leated to mean value)	2	2	2
		range	6	4	4
Occupational status	Distribution	No information (os = 0)	*n* = 3 (5%)	*n* = 3 (20%)	*n* = 3 (20%)
		Employed (os = 1)	*n* = 36 (60%)	*n* = 12 (80%)	*n* = 11 (73.3%)
		Unable to work or on sick leave (os = 2)	*n* = 16 (26.7%)	*n* = 0	*n* = 1 (6.7%)
		Retired (os = 3)	*n* = 3 (5%)	*n* = 0	*n* = 0
		Jobseeker (os = 4)	*n* = 1 (1.7%)	*n* = 0	*n* = 0
		Reintegration (os = 5)	*n* = 1 (1.7%)	*n* = 0	*n* = 0
	Descriptive statistics according to ordinal scales	Median	1	1	1
		Mean	1	0.8	0.9
		SD (leated to mean value)	0.9	0.4	0.5
		Range	5	1	2

*SD* standard deviation, *os* ordinal scale.

The maximum school qualification was classified using the CASMIN classification (Table 1 and Fig. S1). The mean qualification level was 7 (technical college graduation/ high school diploma and vocational training) with a median level of 7 (Table 1 and Fig. S1b), not differing between respective groups. The majority of all subjects had a lower secondary school leaving certificate and vocational training (35% PCS, 27% non-PCS, 33%HC), followed by subjects with a university degree (25% PCS, 53% non-PCS, 20%HC) and with a technical college graduation/ high school diploma and vocational training (18% PCS, 13% non-PCS, 7%HC). The remaining patients had a university of applied sciences degree, secondary school diploma and vocational training or advanced technical college certificate/ high school diploma without vocational training. Most subjects were employed (60% PCS, 80% non-PCS, 73%HC) (Table 1 and Fig. S1c).

### Self-assessment of complaints

Symptoms were reported by 6.7% of subjects in the control group, specifically 12.5% of women (1 out of 8) and 0% of men. No symptoms were reported by the non-PCS group, whereas 100% of the Neurologic PCS group indicated they experienced complaints (Table 2). As part of the self-report questionnaire, subjects were asked to mark those symptoms which they were currently suffering from, based on a list of symptoms. While HC documented an average of *n* = 0.1 symptoms (median 0, range 0–2) and non-PCS group an average of *n* = 0 symptoms, subjects in the PCS group reported an average of *n* = 2 symptoms (median 2, range 1–4).

**Table 2:** Comparison of complaint and symptom frequencies among PCS, NON-PCS, and HC groups.

		PCS	Non-PCS	HC
Number of complaints	No complaints	*n* = 0	*n* = 15 (100%)	*n* = 14 (93.3%)
	One complaint	*n* = 15 (25%)	*n* = 0	*n* = 0
	Two complaints	*n* = 23 (38.3%)	*n* = 0	*n* = 1 (6.7%)
	Three complaints	*n* = 14 (23.3%)	*n* = 0	*n* = 0
	Four complaints	*n* = 8 (13.3%)	*n* = 0	*n* = 0
Exhaustion	Yes	*n* = 29 (48.3%)	*n* = 0	*n* = 0
	No	*n* = 31 (51.7%)	*n* = 15 (100%)	*n* = 15 (100%)
Memory disorder	Yes	*n* = 46 (76.7%)	*n* = 0	*n* = 0
	No	*n* = 14 (23.3%)	*n* = 15 (100%)	*n* = 15 (100%)
Concentration disorder	Yes	*n* = 46 (76.7%)	*n* = 0	*n* = 1 (6.7%)
	No	*n* = 14 (23.3%)	*n* = 15 (100%)	*n* = 14 (93.3%)
Word finding disorder	Yes	*n* = 17 (28.3%)	*n* = 0	*n* = 0
	No	*n* = 43 (71.7%)	*n* = 15 (100%)	*n* = 15 (100%)

In the PCS group, 29 (48.3%) of the patients reported being exhausted, while this symptom was reported by none of the patients in the other groups. Most patients in the PCS group (*n* = 46 [77%]) reported suffering from memory impairment. Only one patient from the HC group and no patient from the non-PCS group reported this symptom. Lack of concentration was reported by 46 (76.7%) of the PCS patients and by one subject from the HC group. The least self-reported symptom was impairment of word-finding. From the PCS group this symptom was reported by *n* = 17 (28%) patients and none of the comparators.

### Neuropsychological test battery

#### PCS patients suffer from significantly more motoric and cognitive fatigue compared to HC and non-PCS

**Fig. 1:**
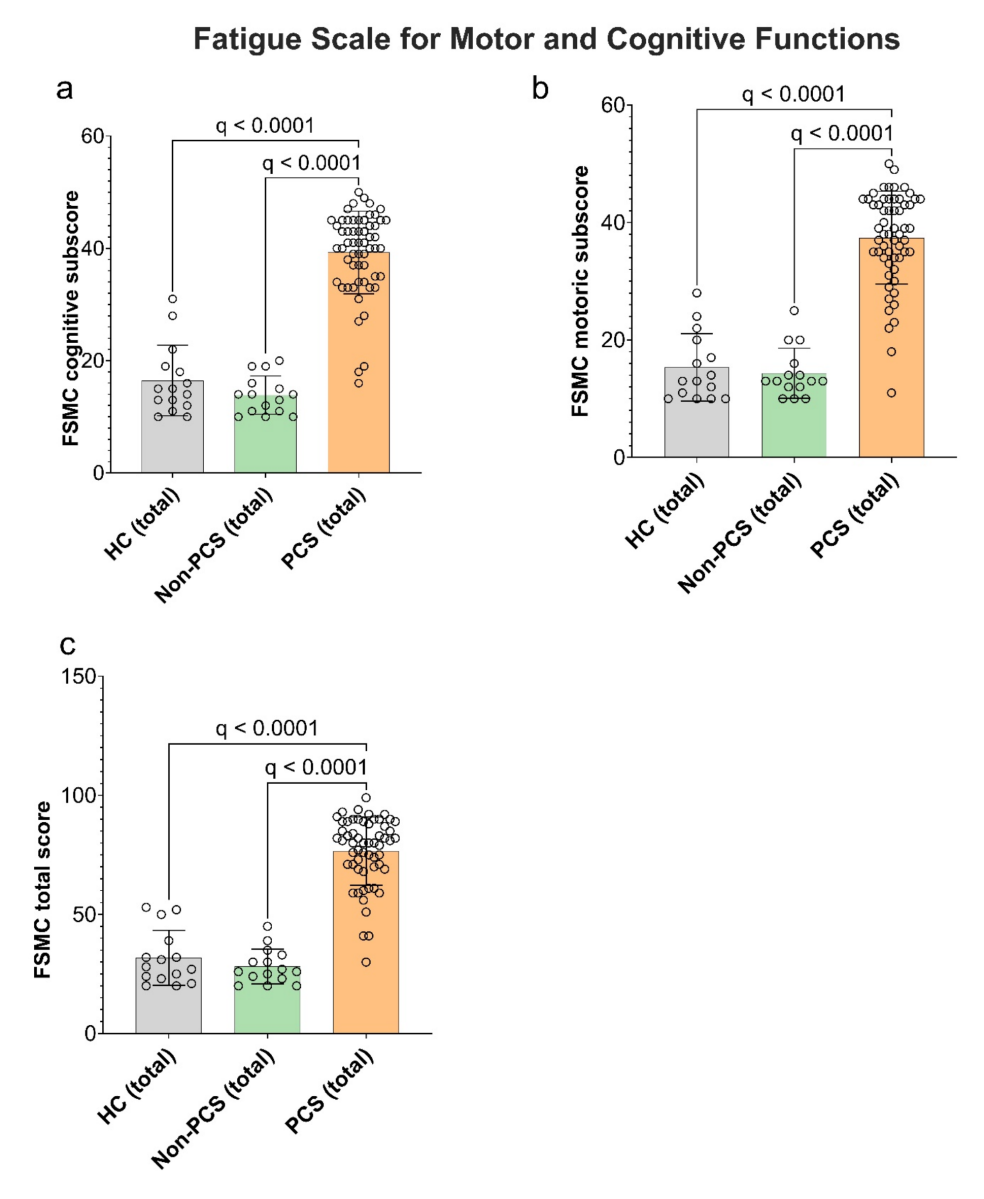
Cognitive and motor fatigue in PCS, non-PCS, and HC. (**a**) Cognitive fatigue, (**b**) motor fatigue, and (**c**) total fatigue are displayed, as assessed using the FSMC. Cut-off values for (**a**) cognitive fatigue are: minor fatigue = 22, moderate fatigue = 27, severe fatigue = 34. Cut-off values for (**b**) motor fatigue are: minor fatigue = 22, moderate fatigue = 27, severe fatigue = 32. (**c**) Cut-off values for total fatigue are: minor fatigue = 43, moderate fatigue = 53, severe fatigue = 63. Data is presented as individual values and as mean ± SD. Data were tested for significance using the Kruskal-Wallis test, followed by multiple comparisons adjusted with the two-stage linear step-up procedure by Benjamini, Krieger, and Yekutieli (BKY). The significance level was set at *q* < 0.05. *PCS* post-COVID syndrome, *non-PCS* non-post-COVID syndrome, *HC* healthy control, *FSMC* fatigue scale for motor and cognitive functions.

Since a high proportion of patients reported being exhausted, we assessed exhaustion using the self-reported *fatigue scale for motor and cognition* (FSMC). The cut-off value for a pathological finding is ≥ 43 for the total score and ≥ 22 for the sub scores. While HC scored a mean of 16 points (median 15, range 10–31) and non-PCS 14 points (median 14, range 10–20) in the cognitive sub-scores, reflecting no cognitive fatigue, PCS patients were suffering from marked cognitive fatigue with an average of 39 points (median 41, range 16–50) which differed significantly from the control groups (PCS vs. HC and non-PCS total q < 0.0001; Fig. 1a). The motoric function documented similar results ((HC average: 15 points (median 13, range 10–28); non-PCS patients 14 points (median 13, range 10–25); PCS patients average 37 points (median 39, range 11–50)), hence significantly higher motoric fatigue in the PCS group compared to HC and non-PCS (Fig. 1b). The total scores reflected the findings of the sub-score analyses (Fig. 1c). A multiple regression identified significant predictors of increased exhaustion: belonging to the PCS group (β = 34.66, *p* < 0.0001, R^2^ = 0.63) and depressive symptoms (HADS-D subscore) (β = 1.05, *p* = 0.02, R^2^ = 0.66) (Table 3). Neither sex nor age had a significant effect.

**Table 3:** Regression analysis FSMC (total score).

Independent covariate	Beta coefficient (β)	95% confidence interval (CI)	*p*-value	*P* value summary	*R* ^2^
PCS-group	34.66	26.27 to 43.04	< 0.0001	****	0.628
Age	− 0.047	− 0.289 to 0.195	0.699	ns	0.409
Male sex	− 2.587	− 8.358 to 3.183	0.374	ns	
HADS-D subscore	1.050	0.161 to 1.94	0.021	*	0.66
HADS-A subscore	0.411	− 0.406 to 1.228	0.319	ns	0.601

#### Increased levels of fatigue are associated with a greater prevalence of depressive and anxiety symptoms

**Fig. 2:**
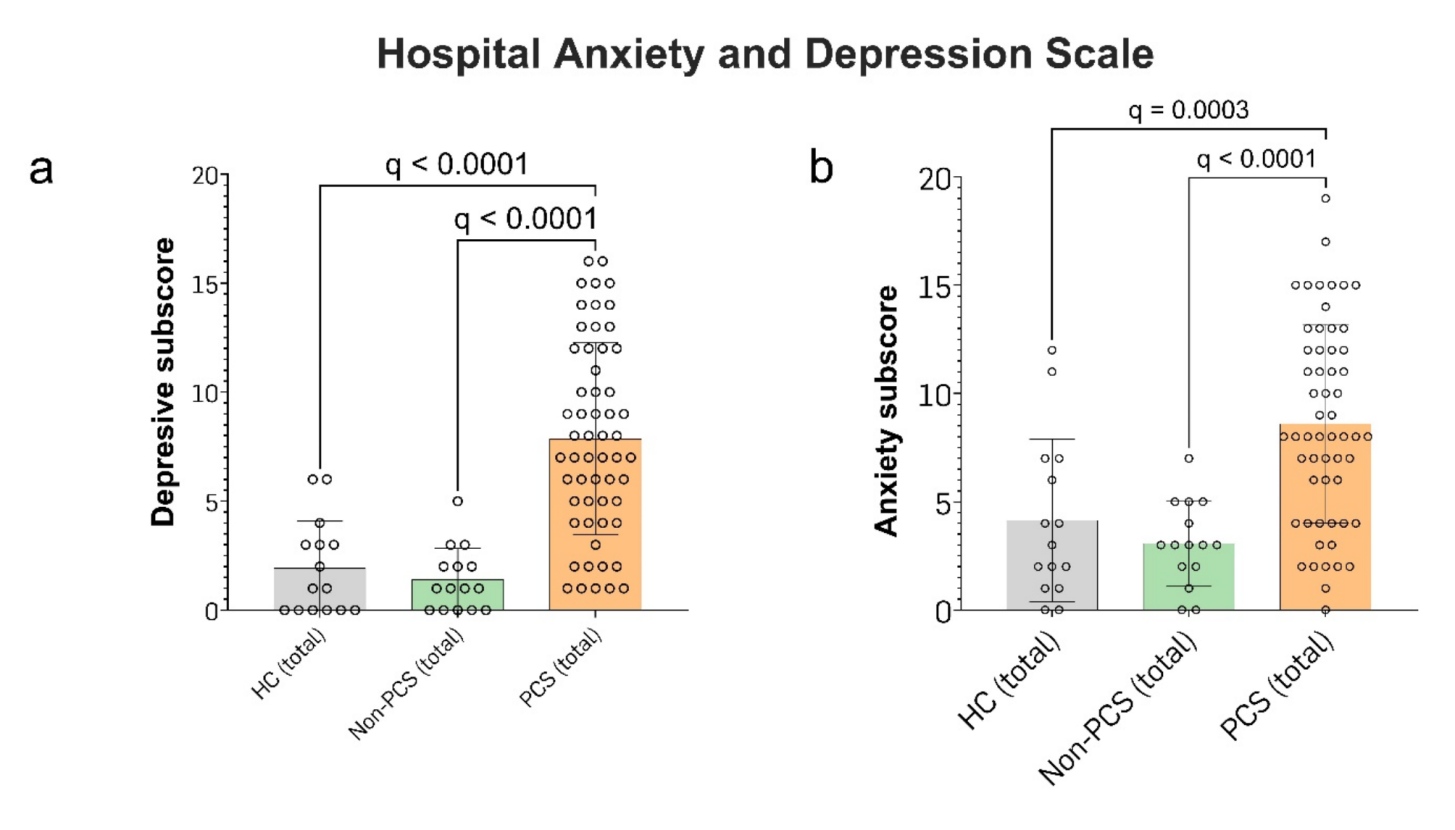
Depressive and anxiety symptoms in PCS, non-PCS and HC. (**a**) Shown is the depressive and (**b**) anxiety subscore, assessed using the HADS-D. Cut-off values are 0–7 = negative, 8–10 = indifferent, > 10 = positive. Data is shown as individual values including the mean +/- SD. Data were tested for significance using the Kruskal-Wallis test, followed by multiple comparisons adjusted with the two-stage linear step-up procedure by Benjamini, Krieger, and Yekutieli (BKY). The significance level was set at *q* < 0.05. *PCS* post-Covid syndrome, *non-PCS* non-Post-Covid syndrome, *HC* healthy control, *SD* standard deviation, *HADS-D* hospital anxiety and depression scale.

To assess depressive and anxiety symptoms we used the German version of the *hospital anxiety and depression scale* (HADS-D). A total score of 8–10 is considered suspicious, a score > 10 is considered conspicuous. HC averaged 2 points (median 1, range 0–6), non-PCS patients 1 point (median 1, range 0–5) and PCS patients 8 points (mean 7, range 1–16), which was significantly higher compared to both HC and non-PCS (q < 0.0001) (Fig. 2a). In the anxiety sub score, PCS patients reached significantly higher scores with 9 points (mean 8, range 0–19) compared to HC (4 points (median 3, range 0–12), q = 0.0003) and non-PCS patients with 3 points (q < 0.0001; Fig. 2b). The regression analysis indicated that the FSMC total score was a significant predictor of both a higher HADS-D subscore (β = 0.12, *p* = 0.0007, R^2^ = 0.81) and HADS-A subscore (β = 0.1, *p* = 0.0077, R^2^ = 0.81) (Tables 4 and 5).

**Table 4:** Regression analysis HADS-D.

Independent covariate	Beta coefficient (β)	95% confidence interval (CI)	*p*-value	*P* value summary	*R* ^2^
PCS-group	0.72	− 2.843 to 4.282	0.688	ns	0.813
Age	0.0366	− 0.0352 to 0.108	0.313	ns	0.390
Male sex	1.123	− 0.588 to 2.834	0.195	ns	0.190
FSMC total score	0.118	0.0517 to 0.185	0.0007	***	0.812

**Table 5:** Regression analysis HADS-A.

Independent covariate	Beta coefficient (β)	95% confidence interval (CI)	*p*-value	*P* value summary	*R* ^2^
PCS-group	− 0.325	− 4.329 to 3.679	0.872	ns	0.813
Age	− 0.0186	− 0.099 to 0.062	0.647	ns	0.390
Male sex	− 0.3076	− 2.231 to 1.615	0.751	ns	0.190
FSMC total score	0.1028	0.0281 to 0.178	0.0077	**	0.8123

#### Assessment of cognitive functions shows fatiguability with impairment of processing speed

**Fig. 3:**
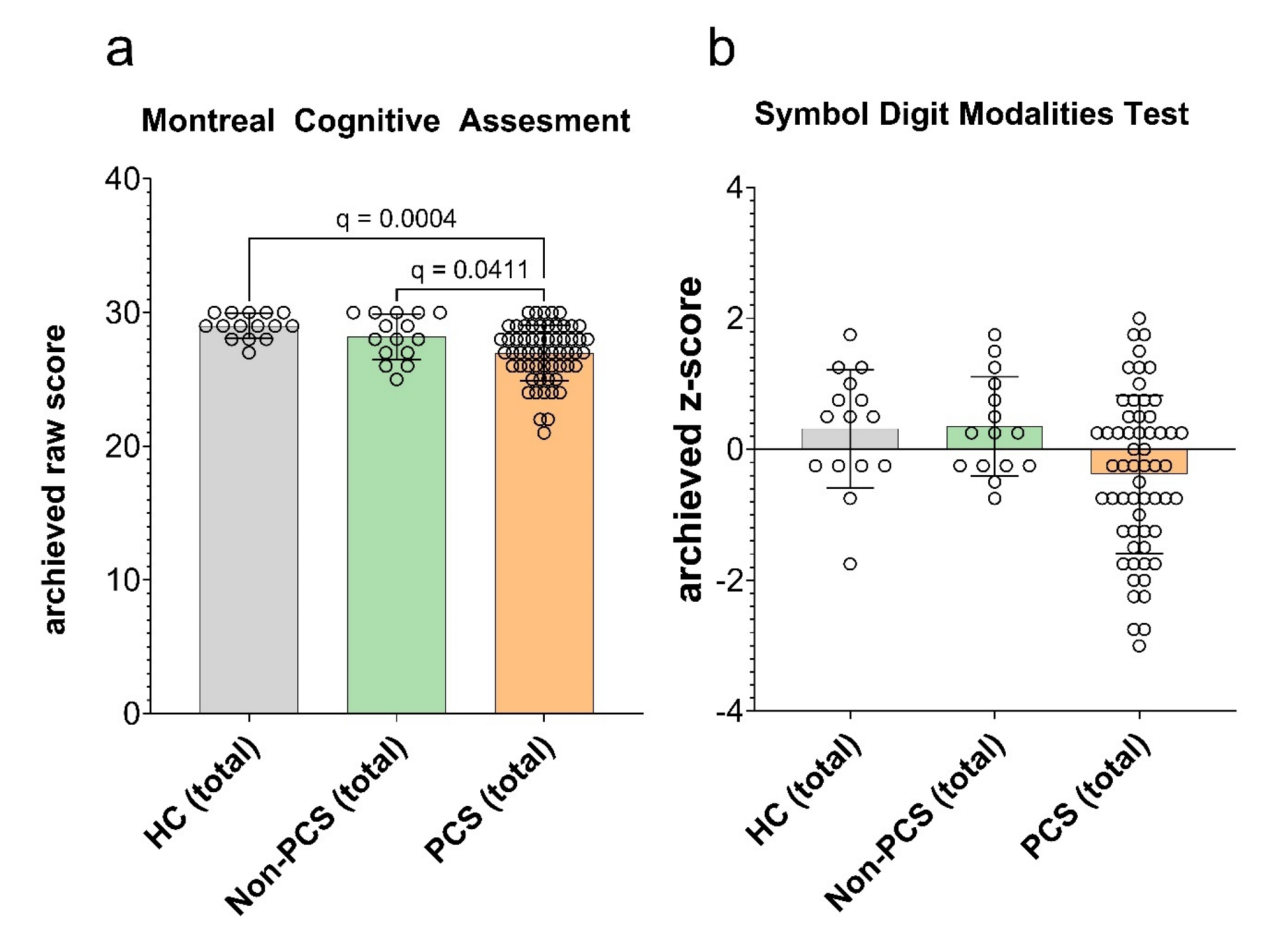
General screening of cognitive deficits and processing speed and concentration in PCS, non-PCS and HC individuals. (**a**) The raw score achieved in the MoCa and (**b**) the result of the SDMT displayed as z-scores are shown. Data is shown as individual values including the mean +/- SD. Data were tested for significance using the Kruskal-Wallis test, followed by multiple comparisons adjusted with the two-stage linear step-up procedure by Benjamini, Krieger, and Yekutieli (BKY). The significance level was set at *q* < 0.05. *PCS* post-Covid syndrome, *non-PCS* non-post-Covid syndrome, *HC* healthy control, *SD* standard deviation, *MoCa* montreal cognitive assessment, *SDMT* symbol digit modalities test.

The general orientation was assessed using the WMS-R orientation questions. There were no relevant differences between the groups with an overall mean value of 14 (median 14, range 13–14) (not shown). A general screening for cognitive deficits was performed using the MoCa. On average, 27 raw score points were obtained in the PCS group (median 27, range 21–30), 28 in the non-PCS group (median 28, range 25–30), and 29 in the HC group (median 29, range 27–30). This was significant between PCS (total) and both control groups (PCS vs. HC: q = 0.0004; PCS vs. Non-PCS q = 0.0411; Fig. 3a). Regression analysis revealed that the prevalence of depressive symptoms, as indicated by the HADS-D subscore, was a significant predictor of reduced cognitive performance, measured by the MoCA score (β = -0.13, *p* = 0.05, R^2^ = 0.68) (Table 6).

**Table 6:** Regression analysis Montreal cognitive assessment.

Independent covariate	Beta coefficient (β)	95% confidence interval (CI)	*p*-value	*P* value summary	*R* ^2^
PCS-group	− 0.893	− 2.537 to 0.750	0.281	ns	0.813
Age	0.002	− 0.031 to 0.036	0.865	ns	0.406
Male sex	− 0.258	− 1.064 to 0.548	0.524	ns	0.223
FSMC total score	0.004	− 0.03 to 0.0376	0.824	ns	0.845
HADS-D subscore	− 0.1334	− 0.264 to − 0.003	0.046	*	0.683
HADS-A subscore	0.01409	− 0.1 to 0.128	0.806	ns	0.594

**Fig. 4:**
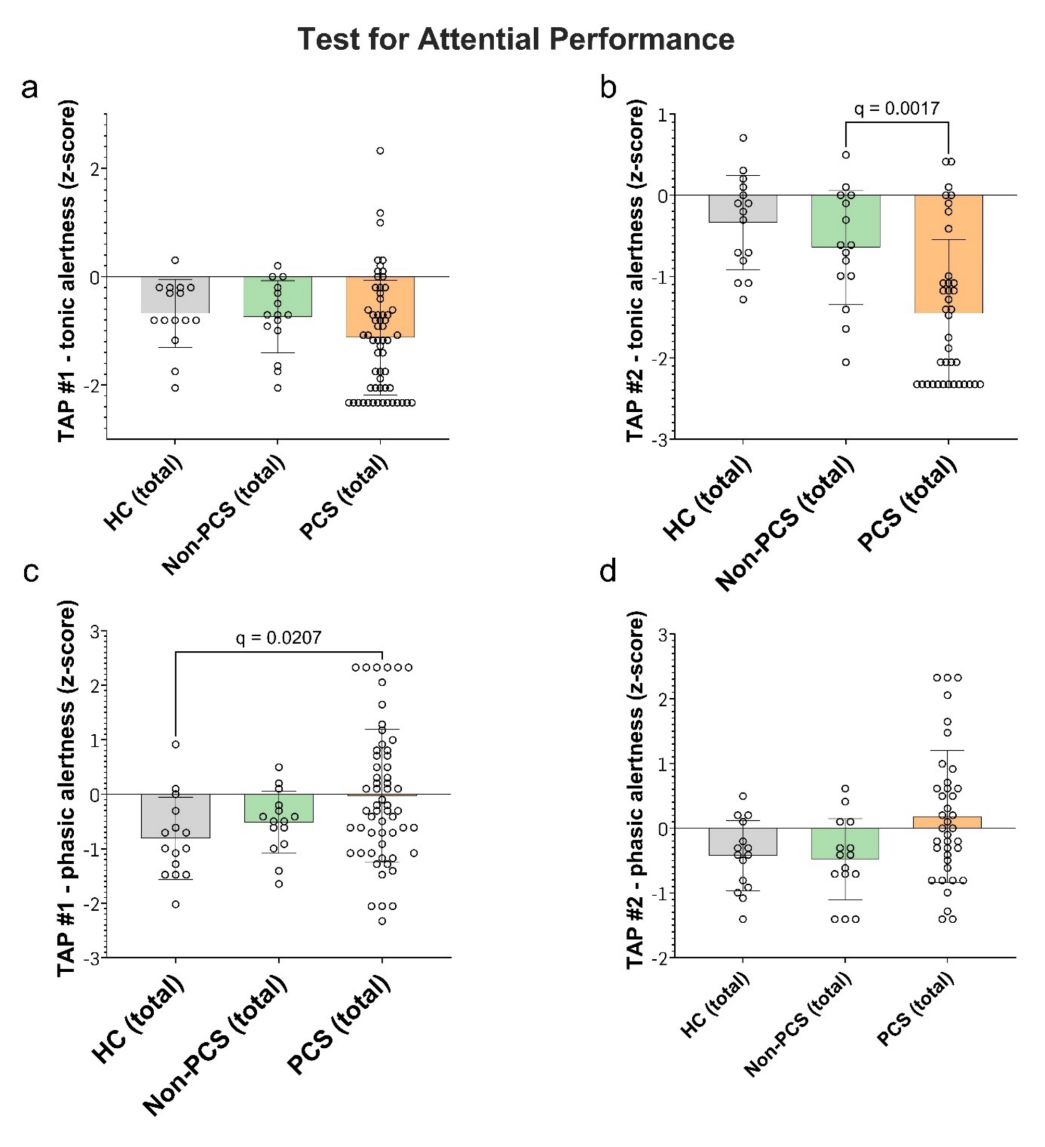
Attentional performance in PCS, non-PCS and HC individuals. The attentional performance of the tested individuals is shown in the form of z-scores, tested using the TAP test at two time points (#1 in (**a**) and (**c**) and #2 in (**b**) and (**d**)) and in two subtests: tonic alertness shown in (**a**) and (**b**) and phasic alertness in (**c**) and (**d**). Data are displayed as individual z-scores with mean +/- SD. Data were tested for significance using the Kruskal-Wallis test, followed by multiple comparisons adjusted with the two-stage linear step-up procedure by Benjamini, Krieger, and Yekutieli (BKY). The significance level was set at *q* < 0.05. *PCS* post-Covid Syndrome, *non-PCS* non-post-Covid syndrome, *HC* healthy control, *SD* standard deviation, *TAP* test for attentional performance.

We assessed information processing speed and concentration using the Symbol Digit Modalities Test (SDMT). A deviation of more than 1.5 standard deviations (SD) below the age norm is considered an indication of a disturbance. The non-PCS group achieved an average SD of 0.4 (median 0.3, range − 0.8 to 2) and HC achieved an SD of 0.3 (median 0.5, range − 2 to 2). The PCS-group scored a mean of -0.4 SD below the age norm (median − 0.3, range − 3 to 2), which was not statistically significant (Fig. 3b). The multiple regression analysis did not reveal any significantly influencing factor (Table 7). Since patients often complain about concentration and attention deficits, we set out to assess general alertness (tonic alertness) and specific responsiveness (phasic alertness) using the subtest *Alertness* of the *Test for Attentional Performance* (TAP) at two different time points. Examining tonic alertness during the first trial, HC achieved a SD of -0.7 (median − 0.8, range − 2.0 to 0.3), non-PCS patients a SD of -0.7 (median − 0.7, range − 2.0 to 0.2) and PCS-patients a SD of -1.0 (median − 1.0, range − 2.0 to 2.0) (ns; Fig. 4a). The second trial was conducted at the end of the test battery to potentially objectify fatiguability over time. HC achieved a SD of -0.3 (median − 0.2, range − 1.0 to 0.7), non-PCS patients achieved a SD of -0.6 (median − 0.6, range − 2.0 to 0.5) and PCS on average a SD of -1.0 (median − 2.0, range − 2.0 to 0.4), which was significantly slower comparing PCS vs. Non-PCS (q = 0.0017) (Fig. 4b).

**Table 7:** Regression analysis SDMT.

Independent covariate	Beta coefficient (β)	95% confidence interval (CI)	*p*-value	*P* value summary	*R* ^2^
PCS-group	0.33	− 0.743 to 1.403	0.541	ns	0.813
Age	0.0001	− 0.022 to 0.022	0.992	ns	0.406
Male sex	− 0.029	− 0.555 to 0.4976	0.914	ns	0.223
FSMC total score	− 0.021	− 0.043 to 0.001	0.065	ns	0.845
HADS-D subscore	− 0.013	− 0.098 to 0.073	0.766	ns	0.683
HADS-A subscore	0.031	− 0.042 to 0.106	0.404	ns	0.594

Examining the phasic alertness during the first trial HC scored on average a SD of -0.8 (median − 1, range − 2.0 to 0.9), non-PCS a SD of -0.5 (median − 0.5, range − 2.0 to 0.5), PCS − 0.03 (median − 0.3, range − 2.0 to 2.0). The difference between PCS and HC was significant with q = 0.0207 (Fig. 4c). This effect was unchanged during the second trial (HC mean SD -0.4 (median − 0.4, range − 1.0 to 0.5), non-PCS SD of -0.5 (median − 0.4, range − 1.0 to 0.6), PCS 0.2 (median 0, range − 1.0 to 0.4) (Fig. 4d). The multiple regression analysis did not reveal any significantly influencing factors (not shown).

Assessment of visuomotor processing speed, working memory and cognitive flexibility was conducted using the Trail Making Test versions A and B. In the TMT-A, HC achieved an SD of 0.3 (median 0.4, range − 0.9 to 1), non-PCS an average SD of 0.2 (median 0.1, range − 2 to 2) and PCS averagely a SD of -0.09 below the age norm (median − 0.1, range − 2 to 2). The TMT-B showed comparable results (HC SD of -0.2 (median − 0.1, range − 2 to 0.7); non-PCS average SD of -0.1 (median − 0.1, range − 2 to 2), PCS averagely a SD of -0.3 below the age norm (median − 0.4, range − 2 to 2) (ns; Fig. S2). The multiple regression analysis did not reveal an any significantly influencing factor (not shown).

#### PCS patients have impaired verbal short-term and working memory as well as word fluency

**Fig. 5:**
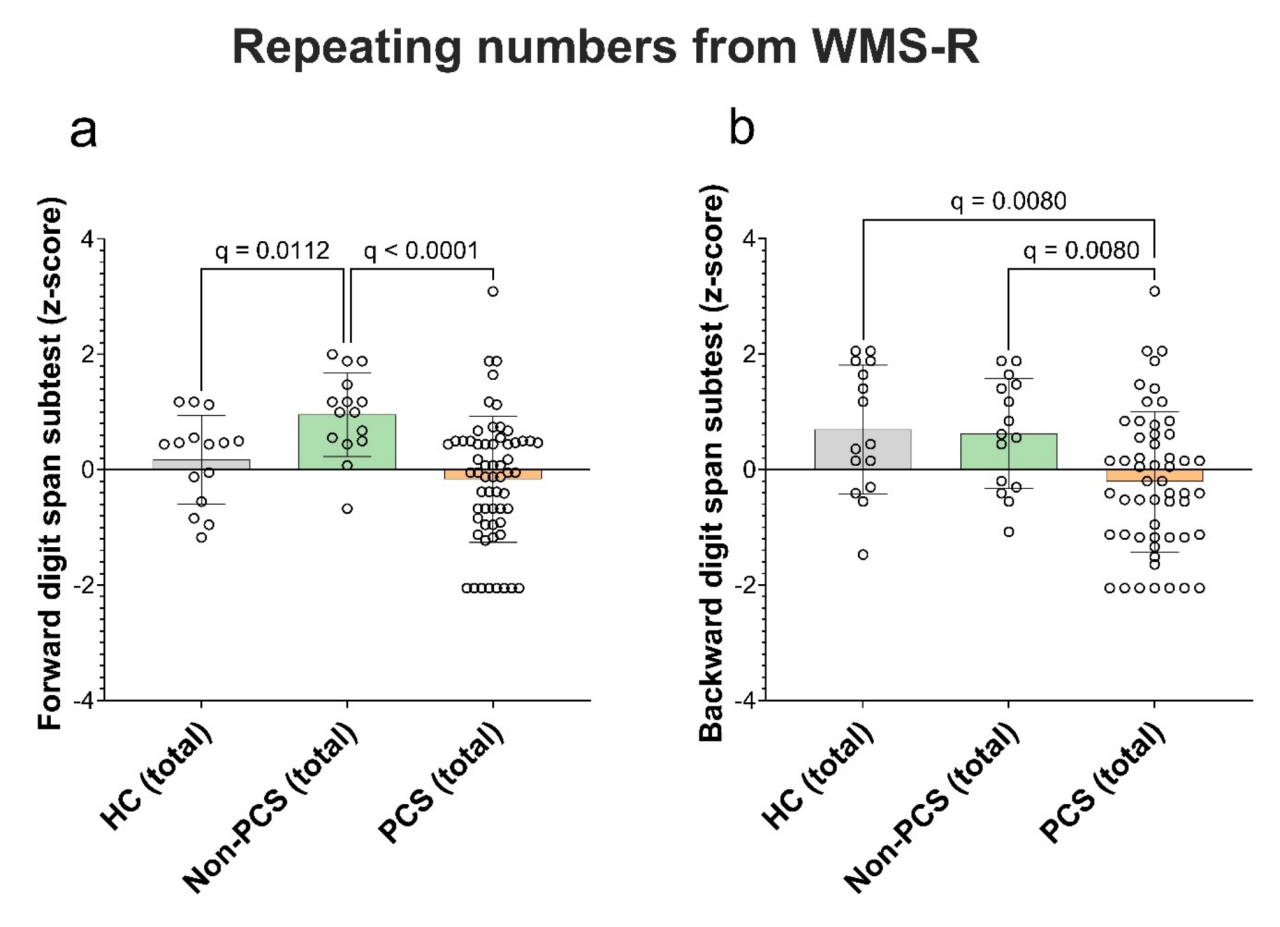
Verbal short-term and working memory in PCS, non-PCS and HC individuals. (**a**) Shown is the achieved z-score in digit span forward subtest and (**b**) digit span backward subtest using “Digit Span” test of the WMS-R. Three columns for each subgroup, divided into female (f), male (m) and total (total). Data are shown as individual values with mean +/- SD. Data were tested for significance using the Kruskal-Wallis test, followed by multiple comparisons adjusted with the two-stage linear step-up procedure by Benjamini, Krieger, and Yekutieli (BKY). The significance level was set at *q* < 0.05. *PCS* post-Covid syndrome, *non-PCS* non-Post-Covid syndrome, *HC* healthy control, *SD* standard deviation, *WMS-R* Wechsler memory test revised.

PCS patients with neurologic symptoms often complain about impairment of word finding and word memory. We assessed verbal short-term and working memory using the digit span forward and backwards test from WMS-R. HC had a SD of 0.2 (median 0.4, range − 1.0 to 1.0), non-PCS achieved a SD of 1.0 (median 1.0, range − 0.7 to 2.0) and PCS-patients achieved on average a SD of -0.2 (median − 0.05, range − 2.0 to 3.0). This was significantly worse in the total PCS group (q < 0.0001) and HC (q = 0.0112) compared to non-PCS (Fig. 5a). In the backward digit span subtest PCS performed significantly worse than Non-PCS and HC (HC SD of 0.7 (median 0.4, range − 1.0 to 2.0); non-PCS SD of 0.6 (median 0.6, range − 1.0 to 2.0), PCS SD of -0.2 (median − 0.3 range − 2.0 to 3.0); PCS vs. HC q = 0.008 and PCS vs. Non-PCS q = 0.008)) (Fig. 5b). The regression analysis revealed that age was a significant predictor of a reduced performance regarding the backward subtest (β = -0.21, *p* = 0.015, R^2^ = 0.41) (Tables 8 and 9).

**Table 8:** Regression analysis WMS-R forward.

Independent covariate	Beta coefficient (β)	95% confidence interval (CI)	*p*-value	*P* value summary	*R* ^2^
PCS-group	− 0.082	− 1.054 to 0.89	0.867	ns	0.814
Age	0.0149	− 0.005 to 0.035	0.14	ns	0.41
Male sex	0.11	− 0.366 to 0.586	0.646	ns	0.224
FSMC total score	0.0009	− 0.019 to 0.021	0.925	ns	0.844
HADS-D subscore	− 0.058	− 0.134 to 0.018	0.134	ns	0.685
HADS-A subscore	0.035	− 0.033 to 0.102	0.306	ns	0.607

**Table 9:** Regression analysis WMS-R backward.

Independent covariate	Beta coefficient (β)	95% confidence interval (CI)	*p*-value	*P* value summary	*R* ^2^
PCS-group	− 2.507	− 10.86 to 5.844	0.5511	ns	0.814
Age	− 0.213	− 0.384 to − 0.042	0.0152	*	0.41
Male sex	1.64	− 2.447 to 5.727	0.426	ns	0.224
FSMC total score	− 0.039	− 0.21 to 0.131	0.6461	ns	0.844
HADS-D subscore	0.044	− 0.607 to 0.695	0.8932	ns	0.685
HADS-A subscore	0.045	− 0.534 to 0.625	0.8761	ns	0.607

**Fig. 6:**
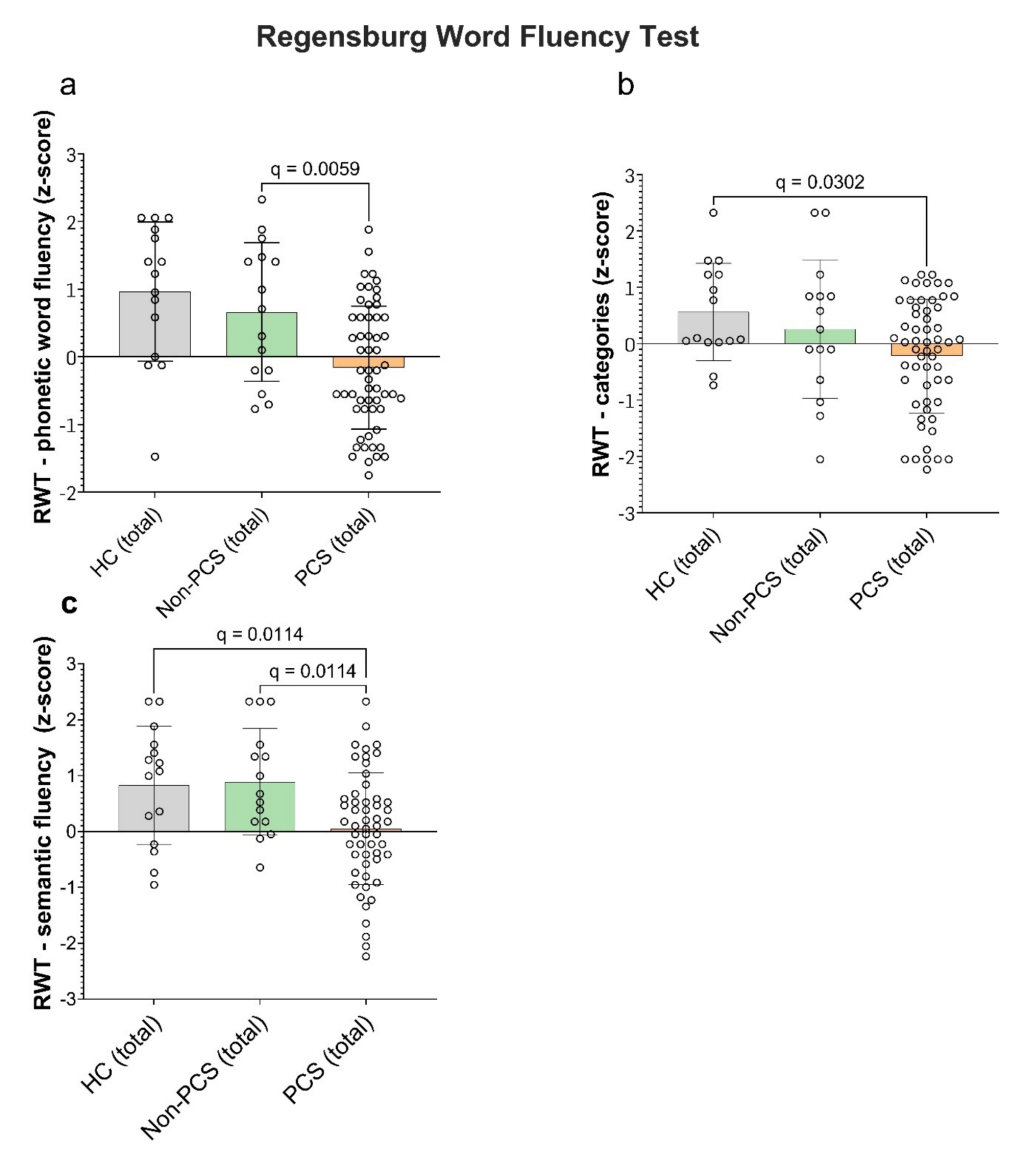
Cognitive flexibility and semantic and phonetic word fluency in PCS, non-PCS and HC individuals. (**a**) Shown are the z-scores in “phonetic word fluency” as well as in (**b**) the subtest “categories” and (**c**) subtest “semantic fluency” using the RWT. Data were tested for significance using the Kruskal-Wallis test, followed by multiple comparisons adjusted with the two-stage linear step-up procedure by Benjamini, Krieger, and Yekutieli (BKY). The significance level was set at *q* < 0.05. *PCS* post-Covid syndrome, *non-PCS* non-Post-Covid syndrome, *HC* healthy control, *SD* standard deviation, *RWT* Regensburg Word Fluency Test.

To assess cognitive flexibility, semantic and phonetic word fluency we used the Regensburger Word Fluency Test (RWT). Regarding word fluency, HC achieved a SD of 1.0 (median 1.0, range − 1.0 to 2.0), non-PCS a SD of 0.7 (median 0.7, range − 0.8 to 2.0) and PCS-patients achieved averagely a SD of -0.2 (median − 0.2, range − 2.0 to 2.0), which was significantly lower for PCS (total) compared to Non-PCS (q = 0.0059) (Fig. 6a). The regression analysis revealed that age was a significant predictor of an improved performance (β = 0.02, *p* = 0.02, R^2^ = 0.42) (Table 10). Results for phonetic category change showed significant differences between PCS and HC (q = 0.0302) (HC with SD of 0.6 (median 0.1, range − 0.7 to 2.0); non-PCS 0.3 (median 0.3, range − 2.0 to 2.0); PCS-patients − 0.2 (median 0.03, range − 2.0 to 1.0) (Fig. 6b). The multiple regression analysis did not reveal any significantly influencing factor (Table 11) In the investigation of semantic word fluency HC achieved a SD of 0.8 (median 1, range − 1.0 to 2.0), non-PCS patients achieved a SD of 0.9 (median 0.7, range − 0.6 to 2.0) and PCS-patients achieved on average a SD of 0.05 (median 0.1, range − 2.0 to 2.0) with a significant difference between PCS and Non-PCS (q = 0.0114) as well as PCS and HC (q = 0.0114) ( Fig. 6c). The regression analysis revealed that age was a significant predictor of an improved performance (β = 0.04, *p* = 0.0025, R^2^ = 0.41) (Table 12).

**Table 10:** Regression analysis RWT word fluency.

Independent covariate	Beta coefficient (β)	95% confidence interval (CI)	*p*-value	*P* value summary	*R* ^2^
PCS-group	− 0.812	− 1.819 to 0.196	0.112	ns	0.816
Age	0.0245	0.004 to 0.0452	0.022	*	0.421
Male sex	0.042	− 0.452 to 0.537	0.864	ns	0.235
FSMC total score	− 0.003	− 0.0236 to 0.019	0.813	ns	0.85
HADS-D subscore	− 0.006	− 0.089 to 0.077	0.892	ns	0.721
HADS-A subscore	− 0.006	− 0.076 to 0.064	0.864	ns	0.608

**Table 11:** Regression analysis RWT categories.

Independent covariate	Beta coefficient (β)	95% confidence interval (CI)	*p*-value	*P* value summary	*R* ^2^
PCS-Group	− 0.745	− 1.863 to 0.374	0.1884	ns	0.816
Age	0.009	− 0.014 to 0.0318	0.4508	ns	0.421
Male sex	0.037	− 0.512 to 0.586	0.893	ns	0.235
FSMC total score	0.005	− 0.018 to 0.028	0.6711	ns	0.85
HADS-D subscore	− 0.009	− 0.101 to 0.084	0.8523	ns	0.721
HADS-A subscore	− 0.041	− 0.118 to 0.036	0.2869	ns	0.609

**Table 12:** Regression analysis RWT semantic.

INDEPENDANT covariate	Beta coefficient (β)	95% Confidence Interval (CI)	*p*-value	*P* value summary	*R* ^2^
PCS-group	− 0.244	− 1.302 to 0.815	0.6469	ns	0.812
Age	0.035	0.013 to 0.057	0.0025	**	0.41
Male sex	− 0.101	− 0.624 to 0.423	0.7019	ns	0.231
FSMC total score	− 0.036	− 0.126 to 0.053	0.4231	ns	0.711
HADS-D subscore	0.015	− 0.059 to 0.089	0.69	ns	0.569
HADS-A subscore	− 0.244	− 1.302 to 0.815	0.6469	ns	0.812

Verbal memory function was investigated using the Verbal Learning and Memory Test (VLMT). The VLMT measures learning, consolidation, and recognition performance. Learning and consolidation did not significantly differ between groups (HC SD of -0.04 (median 0.7, range − 2.0 to 1.0); non-PCS 0.2 (median 0.7, range − 0.9 to 1.0); PCS 0.07 (median 0.2, range − 2.0 to 1.0); ns; Fig. S3a). Recognition performance was also not significantly different (HC achieved an SD of 0.5 (median 0.8, range − 1.0 to 2.0); non-PCS 0.6 (median 0.8, range − 0.5 to 2.0); PCS 0.3 (median 0.3, range − 2.0 to 2.0); ns, Fig. S3b). The overall performance conclusively did not significantly differ (HC SD of 0.8 (median 0.9, range − 0.8 to 2.0), non-PCS patients 1.0 (median 1.0, range 0.3 to 2.0); PCS-patients 0.3 (median 0.7, range − 2.0 to 2.0); ns, Fig. S3c). Regarding the overall performance the multiple regression analysis revealed that having a university degree was associated with better performance in VLMT (β = 0.62, *p* = 0.026, R^2^ = 0.44) (not shown).

Visual memory functions were investigated using the Brief Visuospatial Memory Test (BVMT-R). There was no significant difference between groups (HC SD of 0.8 (median 0.9, range − 0.8 to 2); non-PCS 1.0 (median 1.0, range 0.3 to 2.0); PCS 0.3 below the age norm (median 0.7, range − 2.0 to 2.0); Fig. S4). Regarding the subscore learning and consolidation multiple regression analysis revealed that exhaustion (FSMC total score) was a significant predictor of a reduced performance (β = -0.006, *p* = 0.04, R^2^ = 0.85) (not shown).

## Discussion

Post-Covid is considered a potentially huge burden with massive implications for both the health care system and social economy. Impairment of cognition, in lay media summarized as “brain fog”, is often reported by patients. We here set out to decipher neuropsychological alterations of neurologic PCS patients with potential for a more streamlined therapeutic intervention.

Patients presenting at specialized outpatient clinics most commonly report fatigue, forgetfulness or brain fog^11,16^. This was also evident in neurologic PCS patients investigated in our cohort with significantly higher symptom burden compared to the control groups. Subjects with neurologic PCS felt more exhausted and suffered from memory and impairment of concentration. Moreover, PCS patients scored significantly higher in the HADS questionnaire assessing both depressive and anxiety symptoms. Using a machine learning approach, depressive symptoms as well as cognitive reserve, obesity and change in employment status predicted processing speed and executive function after COVID^17^. We found that PCS patients with neurologic symptoms were impaired in working memory and attention. Of note, we performed the TAP test twice during the neuropsychological assessment revealing significantly longer reaction times at the second investigation, potentially reflecting a consequence of cognitive fatiguability. In line with our findings, in two German and UK cohorts PCS patients showed pronounced cognitive slowing in 53.5% of patients with PCS. PCS response time was more than 2 standard deviations below the mean of the response time of the control group using a simple reaction time test^18^. Besides, neurologic PCS patients investigated here showed impairment of verbal short-term memory, cognitive flexibility and verbal fluency, often reported by patients with difficulties finding words.

Potential reasons for cognitive impairment are manifold. Combined neuropsychological assessment and MRI studies showed that patients with mild COVID with a median follow-up of 79 days after COVID had higher axial diffusivity values in diffusion tensor imaging, suggesting subtle white matter abnormalities after COVID^19^. This might be a consequence of immunological and vascular alterations induced by COVID. Recently, using RNA sequencing immunological alterations in PCS patients could be demonstrated. In a study of patients 12 months after COVID-19 multimodal proteomics analysis showed terminal complement system dysregulation and ongoing activation of the alternative and classical complement pathways^20^. This was associated with increased antibody titers against several herpesviruses. Hence, immune dysregulation with complement activation and thromboinflammatory protein activation might be a driver of PCS. This might also explain long-term cardiovascular outcome following COVID-19 with increased risk of cerebrovascular disorders, dysrhythmias, ischemic and non-ischemic heart disease, pericarditis, myocarditis, heart failure and thromboembolic disease^21^.

Using MRI, Besteher et al. demonstrated that there is a continuum of cortical thickness alterations, most pronounced in PCS patients with cognitive impairment assessed using the MOCA^22^. Additionally, multimodal neuroimaging revealed functional connectivity changes, including bihemispheric hypoconnectivity, reduced grey matter volume, and alterations in white matter. Importantly, grey matter loss was linked to cognitive impairment^23^. Of note, there were also elevated levels of IL-10, IFNγ, and sTREM2 in long-COVID patients, especially in the group suffering from cognitive impairment. Despite the rather small sample size investigated here, we found significantly lower performance in PCS patients compared to HC in the MOCA, a screening instrument for cognitive impairment. Whether long-term risk of cognitive impairment will be increased in PCS patients’ needs further investigation.

There are different therapeutic interventions under investigation. First, prevention is important using vaccination against SARS-CoV-2, since the risk of developing PCS is substantially reduced following vaccination as shown in a population-based study in Sweden with an adjusted hazard ratio of 0.42 (95% confidence interval 0.38 to 0.46)^24^. Recently, the antidepressant vortioxetine showed positive effects in PCS patients in the Digit Symbol Substitution Test in those whose baseline C reactive protein (CRP) was above the mean^25^. Vortioxetine treated patients also showed significant improvement in measures of depressive symptoms and health-related quality of life, presumably due to the antidepressive effect. A post-hoc analysis documented that vortioxetine has positive effects on psychosocial functioning mediated by improvement of fatigue^26^. Since the cohort investigated here also showed higher levels of depressive and anxiety symptoms, positive effects on fatigue might rather be a consequence of the antidepressive effect. Besides, there are several other interventions currently under investigation such as a treatment with melatonin, which stimulates the expression of an intracellular antioxidant^27^, low-dose naltrexone, which may have immunomodulatory effects^28^ or hyperbaric oxygen therapy which is discussed to improve neurocognitive functions and symptoms by inducing neuroplasticity^29^. Further non-pharmacological interventions such as resistance exercises, Pilates, music therapy in combination with cognitive behavioural therapy, and neuromodulation using transcranial direct current stimulation have been explored for post-viral syndromes like PCS, but require more intensive investigation^30^.

Our study has limitations, including its single-center design and the relatively small sample size. Another key limitation is that most of the participants were recruited from our neurological post-COVID outpatient clinic, meaning they primarily had neurological or cognitive issues, making the study population highly selective. This limits the generalizability of our findings to broader groups of people with PCS. Furthermore, the lack of information regarding the comparability of different groups in terms of hospitalization and ICU admission, as well as premorbid comorbidities (particularly neuropsychiatric diagnoses), represents a significant limitation. A strength of the study herein lies in the high quality and broad neuropsychological test battery spanning different cognitive domains with the inclusion of age-matched subjects in the analysis herein.

In summary, our findings suggest that neurologic PCS patients may experience impairments in working memory and attention. However, we did not find evidence that fatigue was a significant influencing factor in the multiple regression analyses. Additionally, neurologic PCS patients tended to report increased symptoms of depression and anxiety, with a possible connection between depressive symptoms and fatigue. It remains unclear whether these issues are linked to a preexisting risk for affective disorders or are a result of the disability caused by the post-COVID condition. Our results may contribute to refining diagnostic procedures and therapeutic approaches for patients with suspected neurologic PCS. Future research should explore immunological and MRI changes alongside neuropsychological assessments to better understand potential diagnostic and therapeutic strategies for this patient group.

## Electronic supplementary material

Below is the link to the electronic supplementary material.

Supplementary Material 1

## Data Availability

Data is provided within the manuscript or supplementary information files. The raw data supporting the conclusions of this article will be made available by the corresponding author upon reasonable request.
