# Supplementary material for: Neurological post-COVID syndrome is associated with substantial impairment of verbal short-term and working memory
Source: Sci Rep. 2025 Jan 11;15:1695. doi: 10.1038/s41598-025-85919-x (PMC11724982; doi:10.1038/s41598-025-85919-x)
Supplement: Supplementary file 1 — Supplementary Material 1 [file 41598_2025_85919_MOESM1_ESM.docx]

**Neurological Post-COVID syndrome is associated with substantial impairment of**

**verbal short-term and working memory**

Jeyanthan Charles James^1^, Hannah Schulze^1^, Nadine Siems^1^, Christian Prehn^1^ , Daniel R. Quast^2^, Nadine Trampe^1^,Ralf Gold^1^ and Simon Faissner^1^

^1^ Department of Neurology, St. Josef-Hospital, Ruhr-University Bochum, Bochum, Germany

^2^ Department of Internal Medicine, St. Josef-Hospital, Ruhr-University Bochum, Bochum, Germany

*Corresponding author*

Prof. Dr. med. Simon Faissner,

Dept. of Neurology, Ruhr University Bochum, St. Josef-Hospital,

Gudrunstr. 56, 44791 Bochum, Germany, E-Mail: [simon.faissner@rub.de](mailto:simon.faissner@rub.de)

**SUPPLEMENT**

**SUPPLEMENTARY METHODS**

The maximum school qualification was classified using the CASMIN classification [S1], with the following ordinal scale: 0= no information, 1= no degree, 2= secondary school diploma without vocational training, 3= secondary school diploma and vocational training, 4= secondary school leaving certificate without vocational training, 5= Secondary school leaving certificate and vocational training, 6= advanced technical college certificate/ high school diploma without vocational training, 7= Technical college graduation/ high school diploma and vocational training, 8= University of applied sciences degree and 9= University degree.

The following neuropsychological test battery was performed:

1. *General Screening of cognitive deficits* – “Montreal Cognitive Assessment” (MoCA) [S2]: The MoCA was developed as a screening instrument for mild cognitive impairment and includes 11 tasks from the cognitive domains of attention, concentration, memory, executive functions, language, conceptual thinking, visuoconstructive skills, arithmetic, and orientation. The execution time is 10-15 minutes. The total test score is used for evaluation, which is a maximum of 30 raw score points. A cut-off value of < 26 raw score points indicates a mild cognitive impairment.
2. *General orientation* – General knowledge and orientation questions from “Wechsler-Memory-Scale-Revised” (WMS-R) [S3]: The purpose of this test is to record the patient's current orientation to the person, time, and place. For this purpose, 14 orientation and knowledge questions are asked, which the patient is to answer immediately. The questions are, for example, "When were you born?" and "What is the name of the current German Chancellor?".
3. *Motor and cognitive fatigue* – “Fatigue Scale for Motor and Cognitive Functions” (FSMC) [S4]: The FSMC is a psychometric procedure that has been validated for assessing fatigue in patients with multiple sclerosis. It is a questionnaire that patients and control subjects must complete independently and without assistance. In total, the test consists of 20 items (10 items each for the subcategories motor function and cognition). The items must be rated on a 5-point likert-scale. The cut-off value for a pathological finding is 43 for the total score and 22 for the sub scores. Further differentiation between mild, moderate, and severe fatigue is also possible based on the scores.
4. *Depressive and anxiety symptoms* – “Hospital Anxiety and Depression Scale” (HADS-D) [S5]: The screening captures symptoms of anxiety and depression. The questionnaire is composed of a total of 14 statements, seven of which represent anxiety symptoms and seven depressive symptoms. For each statement, there are four possible answers scoring from 0 to 3. The most applicable statement within the last week is to be marked. Two independent scores are obtained for the anxiety and depression scales. An example item of the depression scale is: "I look forward to the future with joy" with the answer options "yes, very much"/ "rather less than before"/ "much less than before"/ "hardly at all". A total score below 8 is considered inconspicuous, 8-10 is considered suspicious, a score >10 is considered conspicuous.
5. *Information processing speed and concentration* – „Symbol Digit Modalities Test “(SDMT) [S6]: The SDMT consists of nine abstract symbols, each of which is assigned a number (1-9). The symbols are arranged randomly in nine rows with an empty box attached below each symbol. The respondent or patient must fill as many empty boxes with the corresponding number as possible within 90 seconds. The raw scores obtained are compared with a normative sample, whereby a deviation of more than 1.5 standard deviations (SD) below the age norm is considered an indication of a impairment in the speed of information processing.
6. *Visuomotor processing speed, working memory, cognitive flexibility –* „Trail Making Test “(TMT) [S7]: The test consists of the two parts TMT-A and TMT-B. In TMT-A, 25 pseudorandomized numbers are to be connected as quickly as possible in ascending order. In TMT-B, 13 numbers and 12 letters (arranged in a pseudorandomized manner) are to be connected alternately as quickly as possible in ascending order.
7. *Attention (tonic and phasic alertness)* – Subtest Alertness of the “Test for Attentional Performance” (TAP) [S8, S9]: The TAP is a computer-based test procedure for the differential detection of attention disorders and consists of several subtests. In the context of our investigations, we focused on phasic and tonic alertness at two different time points. Alertness is the ability to develop a readiness to respond to environmental stimuli. Here, tonic alertness is understood as the activation and general readiness to react, while phasic alertness is understood as the ability to increase the readiness to react in response to a certain stimuli, which was in this task an audio warning signal.
8. *Verbal short-term and working memory –* Digit span from the Wechsler Memory Scale- revised (WMS-R) [S3,S10]: The test is designed to assess short-term and working memory abilities and is divided into two subtests. In the forward digit span subtest, the patient is asked to repeat number sequences read out by the test administrator. The difficulty increases from three to eight items per sequence. A maximum of 12 number sequences are repeated, with two sequences having the same number of items. The patient receives one point for each correctly repeated sequence. The test is terminated if two consecutive sequences of the same length are not repeated correctly. In the backward digit span subtest, the patient is asked to repeat number sequences read out by the test administrator in reverse order. The difficulty increases from two to seven items per sequence. Again, a maximum of 12 number sequences are repeated in reverse, and the test is terminated if two consecutive sequences of the same length are not repeated correctly.
9. *Cognitive flexibility, semantic and phonetic word fluency* – phonetic and semantic word fluency and phonetic category change from the “Regensburg Word Fluency Test” (RWT) [S11]: The RWT measures verbal fluency and flexibility as an executive subfunction. To record phonetic word fluency, the patient is asked to generate as many words as possible with a given initial letter within one minute (e.g., "P" words: doll, break, procrastinate, etc.). All German-language words are allowed, except for proper nouns. In addition, no repetitions are allowed, and no words of the same word stem should be listed. To assess mental flexibility to a greater, extend ("phonetic shifting"), the patient is asked to alternately generate words with two different given initial letters within one minute (e.g., "G"-"R" words: green, red, watering can, robber, etc.).
10. *Verbal memory functions* - Verbal Learning and Memory Test (VLMT) [S12]: The VLMT measures verbal memory functions, which are mapped in three parameters: Learning, medium-term memory, and recognition performance. The test consists of a learning list and an interference list, each with 15 semantically independent words, as well as a recognition list with 50 words, containing all words of the learning and interference list, as well as semantically or phonetically similar words. The examiner reads the learning list to the patient. The patient is asked in advance to memorize as many words as possible and, immediately afterwards, to repeat the words (in any order) that he or she was able to remember. The learning list is read out five times and the patient is asked to name all words he or she remembered after each round. After the fifth run of the learning list, the interference list is read out once and the patient is now asked to remember as many words as possible from this list and to repeat them immediately afterwards. Following the interference session, the patient is again asked to repeat the words he or she remembered from the learning list. The words from the learning list are requested again after 30 minutes. The recognition list is then read out and the patient is asked to discriminate for each word with a "yes" or "no" answer whether the word read out was included in the learning list or not.
11. *Visual memory functions* - Brief Visuospatial Memory Test (BVMT-R) [S13]: This test examines visual learning, memory and recognition abilities. During this examination, six different geometric stimuli are presented on a sheet of paper for a period of 10 seconds. The patient is asked to remember the geometric stimuli and their assigned position on the paper. Immediately after presentation, the patient is asked to reconstruct the items and their localization on a blank sheet of paper. Three runs are performed.

**SUPPLEMENTARY REFERENCES**

[S1] Brauns, Hildegard; Scherer, Stefani; Steinmann, Susanne (2003): The CASMIN Educational Classification in International Comparative Research. In: Advances in Cross-National Comparison: Springer, Boston, MA, S. 221–244. Online verfügbar unter <https://link.springer.com/chapter/10.1007/978-1-4419-9186-7_11>.

[S2] Nasreddine ZS, Phillips NA, Bédirian V, Charbonneau S, Whitehead V, Collin I, Cummings JL, Chertkow H. The Montreal Cognitive Assessment, MoCA: a brief screening tool for mild cognitive impairment. J Am Geriatr Soc. 2005 Apr;53(4):695-9. doi: 10.1111/j.1532-5415.2005.53221.x. Erratum in: J Am Geriatr Soc. 2019 Sep;67(9):1991.

[S3] Elwood RW. The Wechsler Memory Scale-Revised: psychometric characteristics and clinical application. Neuropsychol Rev. 1991 Jun;2(2):179-201. doi: 10.1007/BF01109053. PMID: 1844708.

[S4] Penner IK, Raselli C, Stöcklin M, Opwis K, Kappos L, Calabrese P. The Fatigue Scale for Motor and Cognitive Functions (FSMC): validation of a new instrument to assess multiple sclerosis-related fatigue. Mult Scler. 2009 Dec;15(12):1509-17. doi: 10.1177/1352458509348519.

[S5] Hinz, Andreas & Schwarz, Reinhold & Herrmann, Ch & Buss, Ullrich & Snaith, R. (2002). Hospital Anxiety and Depression Scale (HADS-D). Diagnostica. 48. 112-113. 10.1026/0012-1924.48.2.112.

[S6] Sheridan LK, Fitzgerald HE, Adams KM, Nigg JT, Martel MM, Puttler LI, Wong MM, Zucker RA. Normative Symbol Digit Modalities Test performance in a community-based sample. Arch Clin Neuropsychol. 2006 Jan;21(1):23-8. doi: 10.1016/j.acn.2005.07.003.

[S7] Rodewald, Katlehn, Bartolovic, Marina, Debelak, Rudolf et al. (2012) Eine Normierungsstudie eines modifizierten Trail Making Tests im deutschsprachigen Raum. Zeitschrift für Neuropsychologie 23(1): 37–48. [*A norming study of a modified trail making test in German-speaking countries. Journal of Neuropsychology 23(1): 37-48*.] doi: <https://doi.org/10.1024/1016-264X/a000060>.

[S8] Zimmermann, Peter, and Bruno Fimm. "Kapitel 10 Die Testbatterie zur Aufmerksamkeitsprüfung (TAP)." Diagnostik von Konzentration und Aufmerksamkeit (2004): 177. ["*Chapter 10 The Test Battery for Attention (TAP)." Diagnostics of Concentration and Attention (2004): 177.]*

[S9] Dreisörner, Thomas; Georgiadis, Janine (2011): Sensitivität und Spezifität computergestützter Verfahren zur Diagnostik von Aufmerksamkeitsdefizit-/Hyperaktivitätsstörung (ADHS) im Kindes- und Jugendalter. Die Testbatterie zur Aufmerksamkeitsprüfung (TAP) und Testbatterie zur Aufmerksamkeitsprüfung für Kinder (KITAP). Empirische Sonderpädagogik 3 (2011) 1, S. 3-19. In: Empirische Sonderpädagogik 3 (1), S. 3–19. [*Sensitivity and specificity of computerized procedures for the diagnosis of attention-deficit/hyperactivity disorder (ADHD) in childhood and adolescence. The Test Battery for Attention Assessment (TAP) and Test Battery for Attention Assessment for Children (KITAP). Empirical Special Education 3 (2011) 1, pp. 3-19.*] DOI: 10.25656/01:9314.

[S10] Merten, Thomas (2002): Use of the word association technique for memory assessment in older patients. In: Z Gerontol Geriat 15 (1), S. 1–12. DOI: 10.1024//1011-6877.15.1.1.

[S11] Aschenbrenner, S., Tucha, O., & Lange, K. W. Regensburger Wortflüssigkeits-Test: RWT.Hogrefe, Verlag für Psychologie. 2000 [Regensburg Word Fluency Test: RWT. Hogrefe, publisher for psychology]

[S12] Lux, S., Helmstaedter, C., Elger, C. E. (1999) Normierungsstudie zum Verbalen Lern- und Merkfä-higkeitstest (VLMT). Diagnostica 45(4): 205–211. [Standardization study on the Verbal Learning and Memory Test (VLMT). Diagnostica 45(4): 205–211.]

[S13] Penner IK, Filser M, Bätge SJ, Renner A, Ullrich S, Lassek C. Klinische Umsetzbarkeit der kognitiven Screeningbatterie BICAMS bei Patienten mit Multipler Sklerose: Ergebnisse der Machbarkeitsstudie in Deutschland. Nervenarzt. 2021 Oct;92(10):1031-1041. [Clinical practicability of the cognitive screening battery BICAMS in patients with multiple sclerosis: results of the feasibility study in Germany. Nervenarzt. 2021 Oct;92(10):1031-1041.].

**SUPPLEMENTARY FIGURES**


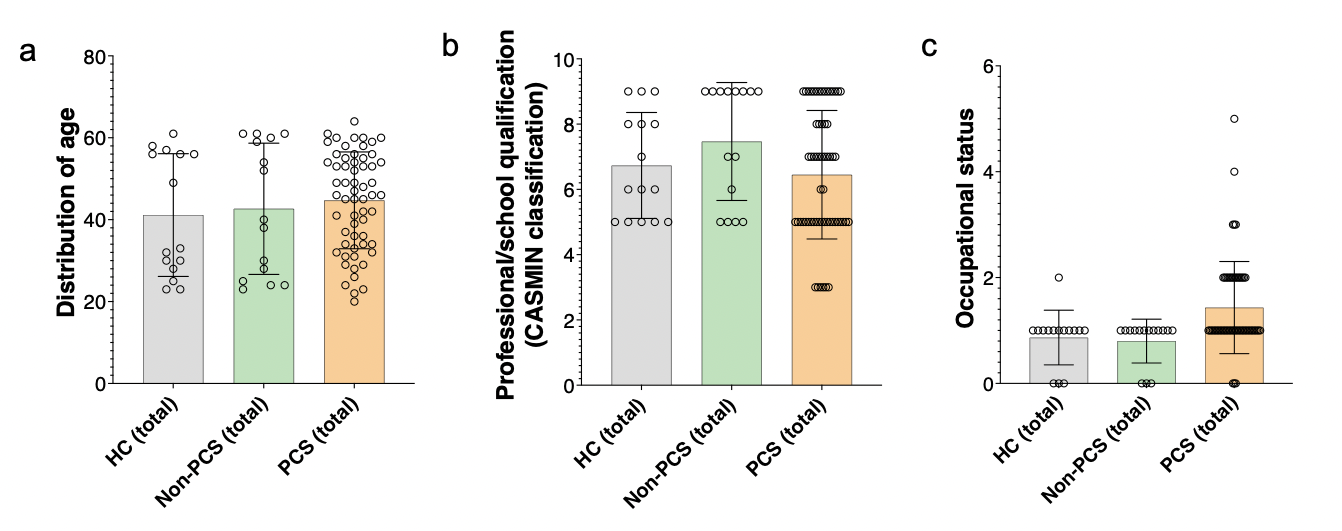


**Figure S1: Sociodemographic characterization and educational status of PCS, non-PCS und HC subjects.** a) Shown is the distribution of age (in years) of the tested subjects. b) Professional and school education according to CASMIN classification. The numbers represent: 0= no information, 1= no degree, 2= secondary school diploma without vocational training, 3= secondary school diploma and vocational training, 4= secondary school leaving certificate without vocational training, 5= Secondary school leaving certificate and vocational training, 6= advanced technical college certificate/ high school diploma without vocational training, 7= Technical college graduation/ high school diploma and vocational training, 8= University of applied sciences degree and 9= University degree . c) Occupational status at time of testing. A distinction is made between 0= no information ,1 = employed, 2 = unable to work or on sick leave, 3 = retired, 4 = jobseeker and 5 = reintegration. Data are shown as individual values including the mean +/- SD. Data were tested for significance using the Kruskal-Wallis test, followed by multiple comparisons adjusted with the two-stage linear step-up procedure by Benjamini, Krieger, and Yekutieli (BKY). The significance level was set at *q* < 0.05. Abbreviations: PCS = Post-Covid syndrome; non-PCS = non-Post-Covid Syndrome, HC = Healthy Control, SD = Standard Deviation, CASMIN = Comparative Analysis of Social Mobility in Industrial Nations.


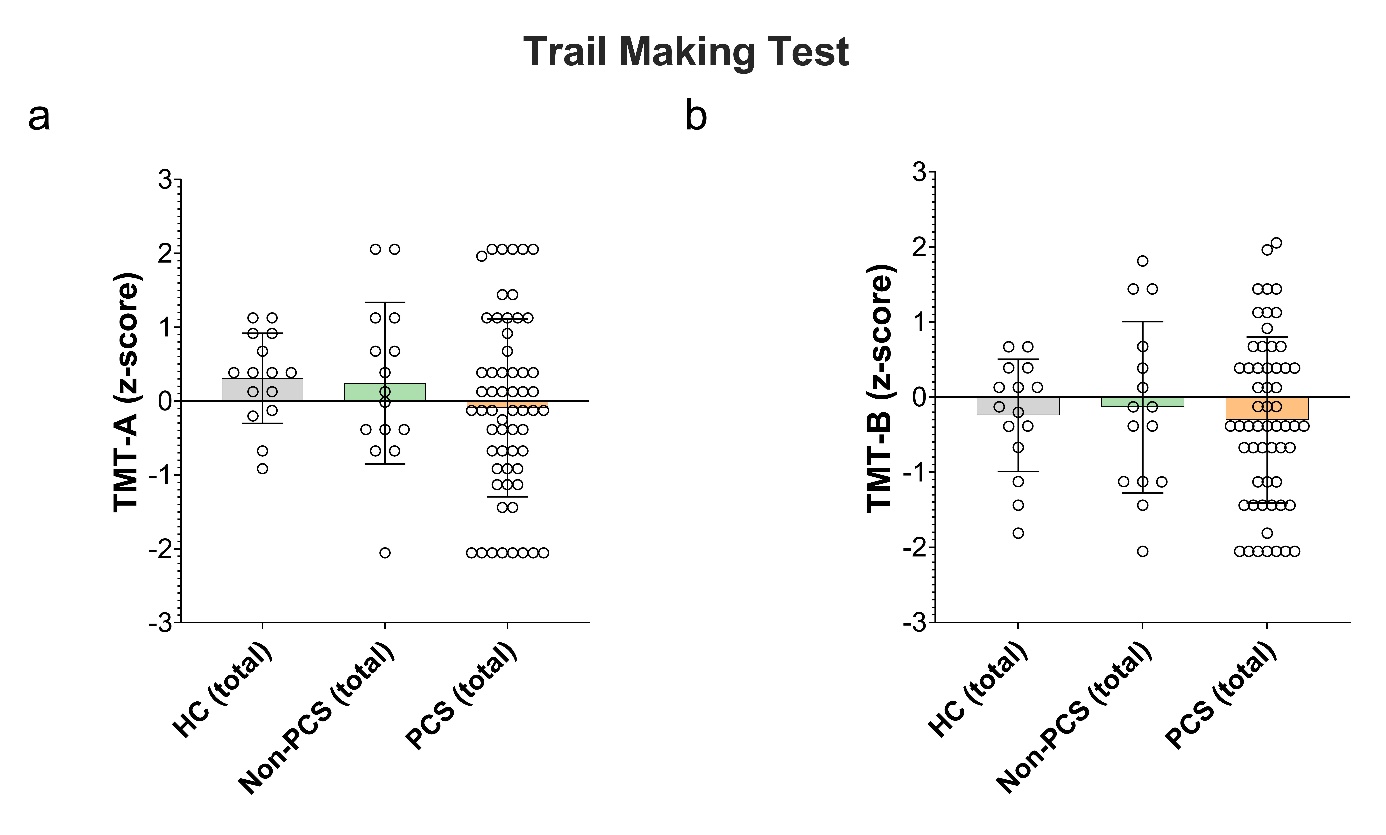


**Figure S2: Visuomotor processing speed, working memory, cognitive flexibility in PCS, non-PCS and HC individuals.** a) Data are shown in form of z-scores of TMT-A and b) TMT-B. Data are shown as individual values including the mean +/- SD. Data were tested for significance using the Kruskal-Wallis test, followed by multiple comparisons adjusted with the two-stage linear step-up procedure by Benjamini, Krieger, and Yekutieli (BKY). The significance level was set at *q* < 0.05.Abbreviations: PCS = Post-Covid Syndrome; non-PCS = non-Post-Covid Syndrome, HC = Healthy Control, SD = Standard Deviation.


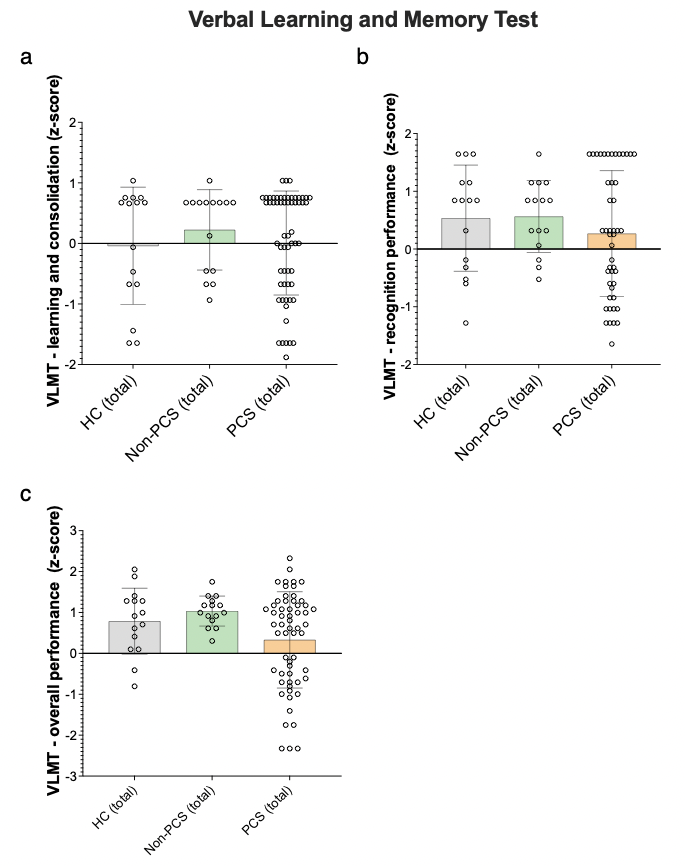


**Figure S3: Verbal memory functions in PCS, non-PCS and HC subjects.** a) The achieved z-scores in the subtest “learning and consolidation” as well as in b) “recognition performance” and c) “overall performance” using the VLMT are shown. Data are shown as individual values with mean +/- SD Data were tested for significance using the Kruskal-Wallis test, followed by multiple comparisons adjusted with the two-stage linear step-up procedure by Benjamini, Krieger, and Yekutieli (BKY). The significance level was set at *q* < 0.05.Abbreviations: PCS = Post-Covid syndrome; non-PCS = non-Post-Covid Syndrome, HC = Healthy Control, SD = Standard Deviation, VLMT = Verbal Learning and Memory Test.


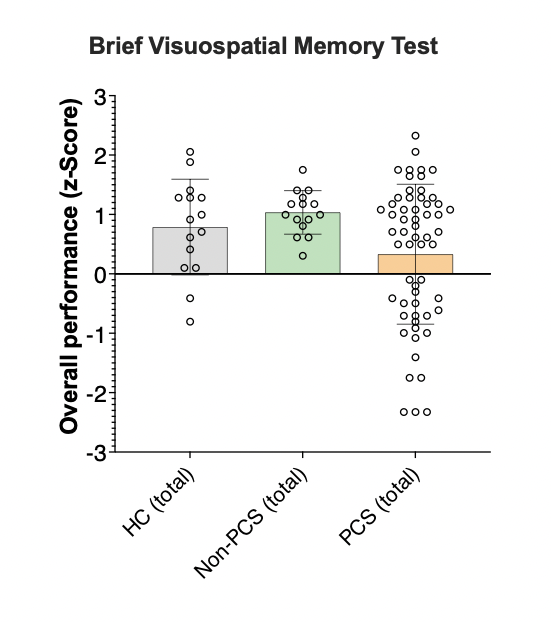


**Figure S4: Visual memory functions in PCS, non-PCS and HC subjects.** The overall performance in the BVMT-R is shown here in form of z-scores. Three columns for each subgroup, divided into female (f), male (m) and total (total). Data are shown as individual values including the mean +/- SD. Data were tested for significance using the Kruskal-Wallis test, followed by multiple comparisons adjusted with the two-stage linear step-up procedure by Benjamini, Krieger, and Yekutieli (BKY). The significance level was set at *q* < 0.05.Abbreviations: PCS = Post-Covid syndrome; non-PCS = non-Post-Covid Syndrome, HC = Healthy Control, SD = Standard Deviation, BVMT-R = Brief Visuospatial Memory Test Revised.
